# ULANC - EUFOREA workshop 2024: bringing the concept of global airways to the frontline of medical care

**DOI:** 10.3389/falgy.2025.1581694

**Published:** 2025-06-30

**Authors:** D. M. Conti, V. Backer, K. Aanaes, A. Azizi, P. W. Hellings, S. Lau, G. Liva, G. K. Scadding, S. Toppila-Salmi

**Affiliations:** ^1^ The European Forum for Research and Education in Allergy and Airway Diseases Scientific Expert Team Members, Brussels, Belgium; ^2^ Escuela de Doctorado UAM, Centro de Estudios de Posgrado, Universidad Autónoma de Madrid. Calle Francisco Tomás y Valiente, n° 2. Ciudad Universitaria de Cantoblanco, Madrid, Spain; ^3^ Department of Otorhinolaryngology, Head & Neck Surgery, and Audiology, Rigshospitalet, Copenhagen University, Copenhagen, Denmark; ^4^ General Practitioner Copenhagen and Ballerup Psychiatric Department, Copenhagen, Denmark; ^5^ Laboratory of Upper Airways Research, Department of Otorhinolaryngology, University of Ghent, Ghent, Belgium; ^6^ Allergy and Clinical Immunology Research Unit, KU Leuven Department of Microbiology and Immunology, Leuven, Belgium; ^7^ Clinical Department of Otorhinolaryngology, Head and Neck Surgery, University Hospitals Leuven, Leuven, Belgium; ^8^ Department of Pediatric Respiratory Medicine, Immunology and Critical Care Medicine, Charité Universitätsmedizin Berlin, Berlin, Germany; ^9^ Department of Otorhinolaryngology, School of Medicine, University of Crete, Heraklion, Greece; ^10^ Department of Allergy & Rhinology, Royal National ENT Hospital, London, United Kingdom; ^11^ Division of Immunity and Infection, University College, London, United Kingdom; ^12^ Skin and Allergy Hospital, Helsinki University Hospital and University of Helsinki, Helsinki, Finland; ^13^ Department of Otorhinolaryngology, Institute of Clinical Medicine, Kuopio University Hospital and School of Medicine, University of Eastern Finland, Kuopio, Finland

**Keywords:** primary care, asthma, CRS, allergy, global airway diseases, comorbidities, ULANC, EUFOREA

## Abstract

The Upper and Lower Airways Northern European Consensus (ULANC) and the European Forum for Research and Education in Allergy and Airways diseases (EUFOREA) organized their first workshop in Copenhagen in January 2024. The aim of the “*Primary care physicians and nurses with an interest in Global Airway Diseases 2024”* was to bring the concept of the global airway to the front line of medical care. ULANC is a multidisciplinary European consortium, that aims to improve the management of comorbid ear, nose and throat (ENT) conditions, such as chronic rhinosinusitis with nasal polyposis (CRSwNP), in patients also suffering from asthma. EUFOREA is an international non-profit organization forming an alliance of all stakeholders dedicated to reducing the prevalence and burden of chronic respiratory diseases through the implementation of optimal patient care via educational, research, and advocacy activities. The inclusive and multidisciplinary approach of ULANC and EUFOREA was reflected in the keynote lectures and workshop faculty coming from the allergology, pulmonology, ENT, and primary health care fields around the central theme of global airway diseases. The current report aims at providing a comprehensive overview of the key statements by the faculty of the “Primary care physicians and nurses with interest in Global Airway Diseases 2024”, allowing all stakeholders in the respiratory field to be up-dated and ready to join forces in Europe and beyond.

## Introduction

Chronic airways and allergic diseases are widespread health concerns that affect millions of people worldwide, not least in the western society, reaching epidemic proportions (1). Conditions driven by type 2 inflammation like asthma, allergic rhinitis (AR), and chronic rhinosinusitis (CRS) can cause significant morbidity, reduced quality of life (QoL), and increased healthcare costs, both directly and indirectly (2). The prevalence of type 2 driven inflammatory conditions has been on the rise globally, with millions of adults and children affected. The role of the primary care physician is critical as a gate keeper for screening, diagnosis and treatment of both upper and lower airways and to refer severe cases to tertiary care (3). Therefore, both frontline healthcare professionals and patients need to better understand type 2 inflammation in order to collectively address the major unmet needs and stop the progression of the disease before it reaches an irreversible chronic state (4).

Both ULANC and EUFOREA are international organisations that form a multi-stakeholder alliance dedicated to reducing the prevalence and burden of chronic respiratory diseases by implementing optimal patient care through education, research and advocacy. The following issues have been identified as barriers to improved care and prevention:

• Lack of collaboration between healthcare professionals at all levels, who may not cover the full complexity of the pathology or may focus on only one segment of the airways
• Lack of collaboration between patients and specialists from different disciplines in the respiratory field.
• Lack of attention to prevention due to a lack of solid knowledge about type 2 inflammatory diseases
• Lack of joint advocacy initiatives to make the voice of patients heard in Europe and beyond.
• Lack of a truly global patient advisory board of patients suffering from chronic long-term respiratory diseases (1, 3).

Based on its' core values of inclusivity and innovation, the “*Primary care physicians and nurses with an interest in Global Airway Diseases 2024”* was organized to bring the concept of the global airway to the front line of medical care, with focus on the theoretical basis, optimal care and a science- based strategic approach to the patient suffering from a type 2 disease or at risk of having it. The collaboration between primary care physicians, pulmonologists, allergologists and ENTs reflects the ambition of both organizations of being inclusive and multidisciplinary.

The *Primary care physicians and nurses with an interest in Global Airway Diseases 2024* reunited four of the most recognized experts from all over the globe to present a total of nine lectures and a plenum discussion and hands on courses on asthma, AR, CRS and their treatment. The meeting aimed to train primary care physicians and nurses in type 2 inflammation, to provide a broad spectrum of pathology, to propose rational therapeutic options, targeted practical tools and to place the patient at the centre of the medical consultation. It was based on five learning objectives: to provide a basic course in airway diseases (asthma, CRS, and allergy); to raise awareness of the concept of global airway diseases in relation to upper and lower airway conditions; to offer insights into the impact of multimorbidity/comorbidities on patients when their conditions are not well-treated or controlled; to identify clear evidence-based guidance for screening patients, including the use of scoring systems in global airway diseases, and determining when to refer patients for upper and/or lower airway conditions to specialist setting; and to develop an understanding of when to refer patients to specialist settings and to recognize the secondary specialists in both upper and lower airway diseases. Lastly, as part of the course all participants were shown how to perform lung function and FeNO, nasal inspection, rhinoscopy, and smell test with Sniffń Sticks 16. Most participants had all tests performed on themselves, excluding rhinoscopy. The full content of this workshop is available on the EUFOREA website (https://www.euforea.eu/events/ulanc-northern-european-masterclass-2024/).

The current report aims at providing a concise overview of the achievements, ambitions, and action plan of ULANC and EUFOREA for the upcoming years, allowing all stakeholders in the respiratory and allergy field to be updated and ready to join forces to tackle the burden of patients affected by chronic respiratory and allergic diseases in Europe and beyond. Overall, the discussions underlined the need to increase the capacity of primary health care staff and train them in the correct recognition of such patients, in order to provide them with appropriate treatment and containment to promote positive change in various areas of chronic respiratory disease and allergy.

### Global airways: Introduction to the pathophysiology and airway symptoms of asthma, allergic rhinitis and CRS

The healthcare system needs to change, we still see paper medical records that are more like an encyclopedia. It is difficult to find the right information. It is in this context that the importance of questioning by properly trained health professionals is crucial (5). The addition of new terminology, with language adapted to the patient, is essential and leads to better communication and understanding of the disease and its correct management (5).

The current/proposed approach is collaborative and considers the global airway perspective (6). Traditionally, specialists only ask about symptoms related to their specialty, but having teams of upper and lower airway specialists in the same practice is the current trend and is of therapeutic benefit to the patient. When the patient expresses a symptom (e.g., cough or secretions), it can be upper or lower airway- related or even gastrointestinal. It should be considered and contextualized, leading the practitioner to think of other or possible associated symptoms, even if they have not yet been reported. The health care system should always suspect associated comorbidities (7), based on standard questionnaires or on the knowledge and experience of clinicians, but it is imperative not to wait for comorbidity to occur or to be diagnosed at a later stage (8).

The phenotypes associated with the global airways are diverse and tell us about variations in disease, likelihood of response to treatment and prognosis. However, there is a common thread that runs through patients with type 2 inflammatory diseases. It is known that someone who is initially diagnosed with asthma has a 58% chance of developing chronic rhinosinusitis with nasal polyps (CRSwNP), an 80% chance of developing allergic rhinitis and a 35% chance of developing atopic dermatitis (9–14). Furthermore, those initially presenting with CRSwNP between 42% and 65% have asthma, as well, however, only half of these patients have a proper diagnosis and treatment, whereas the other half remain undiagnosed and untreated (15, 16). As a screening tool we presented the STARR15 (Standard Test for Asthma, allergic Rhinitis, and chronic Rhinosinusitis) questionnaire, including 4–5 symptoms per disease: allergic rhinitis, CRSwNP and asthma. This should make the investigating physician aware of possible disease in the upper and lower airways (17) which contributes to a worsening of the underlying pathology to a higher level of severity, but also leads to an increase in the complexity of treatment, which has a health cost for the patient and an economic cost for the healthcare system (18–22).

With this in mind, it is of paramount importance to train our first level healthcare providers in interviewing, diagnostic suspicion and co-morbidities using the simple tools available, as well as the latest therapeutic trends. It is important to tailor the consultation to the patient's specific questions and to help the patient understand their condition and what to expect. The presentation or triggers may vary, but correct diagnosis combined with correct patient education is key to effective therapy.

Identifying symptoms (17) and performing the necessary clinical and diagnostic work-up are only the first step, but they must be put into context and should never replace the critical analysis of the treating physician. Especially in respiratory pathology, the concept of diagnostic timeliness becomes important. It is to be expected that patients with asthma will react negatively to appropriate tests when their condition is under control, or that patients with allergic rhinitis will have periods of more or less (or even no) discomfort due to seasonal variability of their disease (23, 24). At present, the Asthma Control Questionnaire (ACQ) is employed in the evaluation of asthma control, enabling the clinician to determine whether a change in score is clinically relevant (25). Similarly, as proposed by the Allergic Rhinitis and its Impact on Asthma (ARIA) guideline, a VAS (Visual Analogue Scale) scale in allergic rhinitis is meaningful for the evaluation of clinically relevant change due to treatment (26). The SNOT-22 (Sino-Nasal Outcome Test) is available for CRSwNP. The articulation of health as the basis for proper patient care must be supported by the state with policies that contribute to the development of national plans (27–30).

Recent studies have shown that 40% of patients presenting to their GP with CRS have asthma and do not know it (31). This highlights the importance of patient education and proper training for our GPs. If these patients are not diagnosed at the earliest stages of care, it may take a long time before they are (16, 31, 32). Perhaps the most important thing is to understand the concept of global airways (33). We know that nasal inflammation affects bronchial inflammation and vice versa (31, 34). It is therefore important to think of the airways as a continuum, affected by the same inflammatory mechanisms and responding in a similar way at all levels (31, 34), although at different time in life. Even in cases where it is claimed that respiratory diseases are not the same disease at different levels, the interaction between upper and lower airway pathology is undeniable (35).

Asthma and CRS are immunological diseases of the bronchi, nose and sinuses. They are inextricably linked as they share the same respiratory tract. Whether due to allergy or an epithelial response to chronic aggression (environmental pollution, particles, smoke or viruses), eosinophil levels will begin to rise (31, 34). This explains why a patient with no history of allergic rhinitis may first develop other respiratory diseases. Patient's exposure must be investigated, but we must also anticipate the progression that typically manifests itself: development of allergic rhinitis, rhinosinusitis, otitis media, esophagitis, among others although not in a certain order and not all of them at the same time in patients, but certainly at group level (31, 34). In general, the presence of respiratory disease with associated comorbidities increases the risk of the disease becoming uncontrolled. This justifies the need for a comprehensive and multidisciplinary approach as well as collaboration.

Endotyping has become a good way to classify respiratory diseases and guide their diagnosis. Many international guides have contributed to this, among which the contributions of the European Position Paper on Rhinosinusitis and Nasal Polyps (EPOS) (36) and the Euforea pocket guides stand out (34, 37, 38, 39).

Among many others, key concepts stand out, such as:

• Defining endotypes helps us in prognosis and in defining therapeutic targets.
• It is important to suspect and identify co-morbidities and to treat type 2 disease as a whole.
• Clinical picture and biomarkers together have predictive potential. They help in prevention and targeted treatment.
• The diagnostic and therapeutic algorithm should be based on the concept of global airways.

### Strategies for a combined approach to CRS and asthma

Airway diseases significantly decrease QoL (31, 34). Often asthma and CRS are the same disease and this can be difficult to assimilate or explain because it is not visible to the patient (33). Having respiratory illnesses is a hidden handicap. A good way to explain it to the CRS patient is that they have asthma in their nose (5).

CRS is among the ten most costly diseases to treat in the US due to the costs associated with, among others, surgery, loss of productivity and medication/treatment (40). If we consider the presence of nasal polyps, their recurrence and asthma, this patient group becomes the costliest to the healthcare system in economic terms (40). One potential issue with these calculations is that patients experiencing an exacerbation of asthma may be operating at a level of 70% efficiency due to shortness of breath. However, this is not necessarily the case, as they may be working despite being sick. Similarly, patients with an exacerbation of CRS may have total nasal blockage and frontal sinus pain, but as this is a chronic disease, they may have been experiencing it for weeks, months or years. The severity of the symptoms may vary, but they do not necessarily require sick leave. However, their working performance may be impaired.

When the patient with CRS presents for consultation at any level of the healthcare system, it is important to make a correct diagnosis based on the latest available evidence. Often, mostly in specialist practices, patients are seen with a long history of treatment, including repeated admissions to the operating theatre (41). It is important to stress that sinus surgery is only one of the therapeutic steps to be taken, in which nasal irrigation, nasal steroids and systemic steroids may also be necessary and part of the treatment (41, 42). Sinus surgery is not expected to resolve the pathology or its comorbidities, but aims to improve the nasal anatomy and condition so that long-term therapeutic continuity can be achieved and the need for operations reduced (43).

In contrast to patients with CRS, the consultation, diagnosis and treatment of asthma patients has become more common at the primary level of the health care system (31, 34). It is not uncommon to find general practitioners (GPs) who have been trained to perform a correct peak flow day-to-day variation, bronchial response to inhaled beta2-agonist (reversibility test) and exercise test, whereas bronchial provocation with mannitol and methacholine is performed in a specialist setting only. This diagnostic and control battery was previously reserved for specialists in the field. At present, the role of each link in the health system in relation to asthma is more clearly defined (Table 1).

**Table 1:** Role of the health-related links involved.

General practitioner	Ear, nose and throat specialist	Pulmonologist
Diagnosis, treatment and monitoring based on the performance of tests such as peak flow and bronchial response to exercise	Treatment of chronic upper airway diseases and ask for bronchial symptoms with referral to pulmonologist in case of suspicion	Diagnosis, treatment and specialized follow-up based on the performance of specific tests such as spirometry, Fractional Exhaled Nitric Oxide (FeNO) test, bronchoprovocation tests and bronchoscopy

There are several tools that contribute to the process of diagnosis and suspicion of comorbidities that are commonly used. Examples include the SNOT-22, VAS and STARR-15. In addition, the most recent international guidelines provided by EPOS (36), the Global Initiative for Asthma (GINA) (44) and EUFOREA (31, 34, 39) are references within the field, simplifying and reducing the burden on the clinician in charge of the consultation. However, all these resources are pointless if they are not accompanied by the appropriate judgement of qualified health care providers.

### Experience as a healthcare provider applied to patient care

Almost half of adults with asthma are unaware that their lung symptoms are due to asthma, according to statistics from Denmark in 2020 (45, 46). The number of respiratory diseases represented by asthma has tripled in adults and children over the last 30 years and the association between asthma and other comorbidities such as rhinitis, sinusitis, gastro-oesophageal reflux and obstructive sleep apnoea has become more evident (45, 46). With these data in mind, it is imperative to take further actions to inform, educate and support patients. Simple measures such as demonstrating correct medication technique, highlighting warning signs or guidelines that should prompt further consultation can make a big difference in their follow-up (1, 3). Encouraging the purchase of a peak flow meter for each patient is another measure that not only contributes to the awareness of one's own disease, but also provides a new parameter by which the patient can become aware of his or her general condition (31, 34).

No healthcare system is perfect, and many face similar challenges. On a daily basis, GPs are confronted with a lack of resources, a lack of time to invest in an individualised consultation, and patients who are unaware of the importance of taking their medication correctly and regularly and monitoring their condition (47). All this makes it difficult to generate therapeutic engagement. This reality contrasts with what is expected of a first level health care provider, such as being a referent in communication with the patient, being able to educate and guide them along the therapeutic pathway, implementing lifestyle changes that contribute, and above all fulfilling the role of providing an accurate diagnosis and appropriate treatment (47, 48). The latter is essential to avoid worsening of the current condition and the development of associated comorbidities.

GPs are often the first point of contact for people with respiratory symptoms. They carry out the initial assessment, make the initial diagnosis and prescribe standard medications to initiate treatment with the goal of disease control (47, 48). The specialist's role begins when an advanced diagnosis is needed, a review of current treatment is required or special circumstances arise (34, 37–39). This organisation of the health system appears to be optimal, as it allows a greater number of patients to be reached at primary level, reduces hospital congestion and prioritises specialised care for those who really need it (34, 37–39). It has been shown, that a substantial part of patients with severe asthma, indicated by their level of treatment is still taken care of in primary care (49), and as for now with the new biologics these patients would gain by being referred.

There are many reasons for referral from the first to the second level of care. Some of these are atypical or progressive presentation, the presence of alarming symptoms such as fever, pain, one-sidedness, hoarseness and dysphagia, the assessment of occupational symptoms, pregnancy or suspicion of neoplasia (31, 34, 37–39). In general, it is recommended when the patient is not improving despite appropriate treatment or when it is felt that the proposed treatment or even the underlying diagnosis should be evaluated (31, 34, 37–39). With this in mind, a large part of this workshop was devoted to training and imparting basic practical knowledge and skills, with the aim of providing a theoretical basis and delving into a number of practical tools that are critical in everyday medical practice. These sessions were divided into three groups, as shown in Table 2.

**Table 2:** Theoretical and practical skills applicable to the health context.

Hands-on crash course	Breakout sessions	Case-based sessions on asthma and CRS
Nasal inspection (anterior rhinoscopy+fiber endoscopy)	Practical application of acquired knowledge	Clinical case presentations
FeNO and EOS measurement	Views according to health system level	Evaluation and discussion of diagnostic options
Lung function measurement	Practical experience vs. theoretical knowledge	Therapeutic options
Smell test 16 Sniffiń sticks	Screening strategies and counselling	What to expect and how to proceed?
Questionnaires of global airways		When and how to refer?

### Use of monoclonal antibodies in the treatment of asthma and CRS

The approach to airway pathology should be multidisciplinary (33, 35). Both the initial consultation with the GP and any necessary referral to ENT and/or pulmonology services should be articulated (33, 35). Nowadays, it is more common to find joint consultations between the above services, with each specialist focusing their questioning on their area of expertise, without neglecting the rest of the airway (50). The result is a comprehensive approach that considers the airway as a continuum, benefiting the patient in terms of diagnosis, the health system in terms of economics, and the clinicians involved in terms of time spent per patient (50).

The management of asthma has evolved over time. Much has been done to arrive at the current approach of Assessment, Adjustment and Review (44). The premise is based on making a correct diagnosis, which then needs to be reviewed and adjusted over time due to the variable nature of the pathology (31, 34). This is also reflected in the results of asthma tests, which are not consistently the same. Therefore, the diagnosis of asthma needs to be sought and each test needs to be contextualised (31, 34).

Biologics have emerged in recent years as a novel approach based on the interruption of pro-inflammatory signalling. There are currently six in the therapeutic armamentarium: Omalizumab, Mepolizumab, Reslizumab, Dupilumab, Benralizumab, and Tezepelumab (Table 3). All of the aforementioned biologics have been indicated for patients with asthma. However, currently, only three of them have been approved for the treatment of CRSwNP: omalizumab, mepolizumab and dupilumab (31, 34). While they are considered one option among many, it is undeniable that perhaps their greatest contribution has been to dramatically reduce the need for prolonged courses of corticosteroids, reduce hospitalisations, reduce the need for revision FESS surgery and improve lung function (51–53). There is currently no consensus on the best time to stop treatment with biologics. This is due to individual patient response, but more importantly the variable nature of the pathology (31, 34, 50, 52, 53).

**Table 3:** Biologics indicated for the treatment of asthma.

Molecule	Target	Indications	Clinical effects	Effect on biomarkers	Predictors of treatment response
Omalizumab	IgE	Severe asthma and exacerbations or daily OCS use	Reduce exacerbations	No current available biomarkers	Allergic driven disease
		Perennial atopy combined with allergen induced symptoms	Reduce OCS use		Atopy (positive SPT/specific IgE)
		Total IgE within dose range	May improve lung function and symptoms		Exacerbations
					Childhood onset asthma
					FeNO ≥20ppb
Mepolizumab	IL-5	Severe asthma and exacerbations or daily OCS use	Reduce exacerbations	Reduce eosinophils	High blood eosinophils
			Reduce OCS use		Exacerbations
			May improve lung function and symptoms		Adult onset asthma
					Nasal Polyposis
Reslizumab	IL-5	Severe asthma and exacerbations or daily OCS use	Reduce exacerbations	Reduce eosinophils	High blood eosinophils
			Reduce OCS use		Exacerbations
			May improve lung function and symptoms		Adult onset asthma
					Nasal Polyposis
Dupilumab	IL-4R/IL-13	Severe asthma and either:	Reduce exacerbations	Reduce FeNO	High blood eosinophils
		Exacerbations and increased eosinoplils or FeNO	Reduce OCS use	Reduce IgE	High FeNO
		or daily use of OCS	Reduce nasal polyposis	Increase blood-eosinophils	Exacerbations
			May improve lung function and symptoms		Nasal polyposis/atopic dermatitis
Benralizumab	IL-5R*α*	Severe eosinophilic asthma	Reduce exacerbations	Reduce eosinophils	High blood eosinophils
			Reduce OCS use		Exacerbations
			May improve lung function and symptoms		Adult onset asthma
					Nasal Polyposis
					Higher levels of FEV1
Tezepelumab	TSLP	Severe asthma	Reduce exacerbations	Reduce multiple T2	Exacerbations
			Improve FEV1	inflammatory biomarkers	Lung function
			Reduce the disease symptom score		FeNO levels

FeNO, fraction Of exhaled nitric oxide; EOS, eosinophils.

Focusing on CRSwNP, a 3-fold increased likelihood of recurrence has been observed in patients with eosinophilic inflammation (54, 55). Diagnosis and follow-up are based not only on clinical findings, but also on the practical support provided by the use of nasal endoscopy and tools such as the Nasal Polyp Score (NPS) (56).

It is important to emphasise that, especially in CRS patients, there is no single therapeutic intervention that can alone resolve the disease (57). It must be seen as a pathology with a variable course for which the patient must follow a therapeutic pathway (58). In this step-by-step process, there will be basic first-order or maintenance interventions on which to build and from which to progress in complexity (59). EUFOREA has proposed an intuitive guide that can be understood and followed by health professionals at all levels, which takes into account the context of the patient and their pathology, and proposes the therapy that is most appropriate at that stage. It then progresses in terms of complexity, taking into account the patient's clinical markers (39).

The use of biologics in CRSwNP is the last link currently available and has inclusion criteria that vary between countries due to cost and the capabilities of each health system. To give an example, in the case of Denmark, the patient must meet all the major criteria (bilateral polyps, ESS within three years, type 2 inflammation and topical treatment >3 months) and at least 3 of the 5 minor criteria (systemic corticosteroids, SNOT-22 ≥ 50, anosmia tested with Sniffin' Sticks between 0 and 8, NPS ≥5 and asthma treated with inhaled corticosteroids). Once again, EUFOREA has made an effort to establish clinical criteria (39, 56–59).

The role of endoscopic sinus surgery remains and has been reassessed. Its potential is decisive if it is used in context and as one of the tools in the arsenal. Its major indication is based on those cases where the sinus anatomy and the sinus context do not allow an adequate access for treatment or where the inciting allergen resides in the sinuses (allergic fungal sinusitis) (39).

### Summary

The *Primary care physicians and nurses with an interest in global airway diseases 2024* offered a unique perspective on prevention, diagnosis and treatment, with a focus on understanding when to refer patients to specialist settings and recognising the secondary specialists in both upper and lower airway diseases. Gaining insight into the impact of multimorbidity/comorbidities on patients when their conditions are not well-treated or controlled, and gaining evidence-based guidance for screening and referral to specialist settings are concepts that have been incorporated into the ULANC - EUFOREA portfolio of activities for 2024 and beyond.

## Data Availability

The original contributions presented in the study are included in the article/Supplementary Material, further inquiries can be directed to the corresponding author/s.
